# Using Participatory Learning and Action in a Community-Based Intervention to Prevent Violence Against Women and Girls in Mumbai’s Informal Settlements

**DOI:** 10.1177/1609406920972234

**Published:** 2020-11-24

**Authors:** Proshant Chakraborty, Nayreen Daruwalla, Apoorwa Deepak Gupta, Unnati Machchhar, Bhaskar Kakad, Shilpa Adelkar, David Osrin

**Affiliations:** 1 Program for Prevention of Violence against Women and Children (PVWC), Society for Nutrition, Education and Health Action (SNEHA), Mumbai, Maharashtra, India; 2 School of Global Studies, 221009 University of Gothenburg, Sweden; 3 Institute for Global Health, 204274 University College London, United Kingdom

**Keywords:** community participation, domestic violence, gender-based violence, intimate partner violence, participatory learning and action, poverty areas, Mumbai, India

## Abstract

For over 3 decades, participatory learning and action (PLA) techniques have been prominent in formative and evaluative studies in community-based development programs in the Global South. In this paper, we describe and discuss the use of PLA approaches at the beginning of a community-based program for prevention of violence against women and girls in Mumbai’s urban informal settlements. We adapted six PLA techniques as part of a formative community mobilization and rapid needs assessment exercise, addressing perceptions of violence prevalence, sources of household conflict, experiences of safety and mobility, access to services, preferences for service and support, and visualization of an ideal community free from violence. We describe the collaborative process of developing and implementing PLA techniques and discuss its relevance in generating contextual and grounded understandings of violence as well as in identifying factors which can potentially enable and constrain interventions.

## Introduction

Participatory approaches have been widely used in social science and applied research since the 1940s, as scholars and researchers engaged with participants, applied scientific knowledge to bring about solutions for local problems, and were driven by ethical concerns and responsibilities and questions of justice (Chambers, 1994a, 2005; Kenton, 2014; Kindon et al., 2007; Manzo & Brightbill, 2007; Whyte et al., 1989). Such approaches were collectively termed participatory action research (PAR), which emphasized the importance of an “emic” perspective, close attention to context, and recognition that local communities were aware of the problems they faced and the solutions that were required to alleviate them (Chambers, 1994a; Kindon et al., 2007; Whyte, 1995).

Such participatory processes included rapid rural appraisal (RRA) and participatory rural appraisal (PRA), involving scholars, government officials, non-governmental organization (NGOs) and rural communities (Chambers, 1994a, 1994b, 1994c; Kenton, 2014). In India, the 73rd Constitutional Amendment Act was passed in 1993, giving constitutional status to Panchayati Raj institutions—the basic self-governing unit in Indian polity at the village, block, and district levels (Singh, 1994). The 73rd Amendment encouraged rural groups to participate actively with the state and other non-governmental actors to undertake local development projects using PRA (Mascarenhas & Kumar, 1991). From the 1990s onward, participatory methods were adapted for non-rural contexts and issues such as poverty, malnutrition, urban governance and policy, livelihood, development and monitoring and evaluation (Abbot, 1999), and were termed “participatory learning and action” (PLA).

PLA approaches have been adapted and promoted by development and health agencies, especially in maternal and newborn healthcare (Gope et al., 2019; Prost et al., 2013; Seward et al., 2017). The World Health Organization (WHO, 2014) and the Indian National Health Mission (NHM, 2018) have endorsed the use of PLA to improve the health of women and children. PLA techniques have also been used to engage multiple stakeholders and recipients in collaborative primary health projects with migrant communities in Ireland (de Brún et al., 2017; O’Reilly-de Brún et al., 2016), prevent violence against women and girls through women’s groups and health activists in rural India (Nair et al., 2020), empower sex workers in Cambodia (Busza & Schunter, 2001), and decolonize methodologies and examine power and privilege in higher education institutions in post-apartheid South Africa (Bozalek & Biersteker, 2010). However, critics have raised cautions about the adoption of participatory approaches, especially questions over whose knowledge counts in terms of social domination and unequal gender relations (Mosse, 1994).

In this article, we discuss our experience of using PLA in a community-based intervention to prevent violence against women and girls in Mumbai’s urban informal settlements. We present evidence from a formative community mobilization process that incorporated six key PLA techniques and was conducted in 24 clusters that comprised the intervention arm of an ongoing cluster randomized controlled trial (Daruwalla et al., 2019b). While we have had previous experience of using PLA techniques in rapid community needs assessment (Daruwalla & Prevention of Violence against Women and Children, 2012), the issue of violence against women and girls posed certain challenges because of its sensitive nature and women’s reticence to disclose abuse and seek help. For instance, according to the Fourth Indian National Family Health Survey (NFHS-4), only 14% of women who have faced physical or sexual violence have sought help, which has declined from 24% reported in NFHS-3 (International Institute for Population Sciences [IIPS] & ICF, 2017, p. 572).

However, our experience here shows that using PLA tools as part of formative community mobilization is a sustainable, low-technology and labor-intensive process to establish relationships between NGO workers and community members prior to the rollout of interventions. Our findings suggest that adapting PLA techniques to the issue of violence against women and girls can elicit contextual evidence on the status of women and girls, gender relations and their experiences of violence and discrimination. Finally, participatory approaches can help in evaluation design and identification of causal pathways and potential mechanisms for change in programs.

## Research Context

According to the World Health Organization, one-third of women globally have faced physical or sexual violence in their lifetime (WHO, 2013). Violence and abuse adversely affect physical, emotional and mental health and wellbeing of women, children, and communities (WHO, 2013), particularly those who live in vulnerable conditions of poverty, dispossession, conflict, and structural violence (Crooms et al., 2011; Montesanti, 2015). The NFHS-4 found that 29% of women had faced physical or sexual domestic violence (IIPS & ICF, 2017). The 2014 *Lancet* series on violence against women and girls presented global evidence on programs that are successful in preventing violence against women and girls. Programs that engaged multiple stakeholders and addressed risk factors such as unequal social norms were found to be successful in preventing violence (Ellsberg et al., 2015). Such programs adopted socio-ecological approaches (cf. Heise, 1998) and used gender transformative and intersectional gender power analysis to promote collective action (Michau et al., 2015). Similarly, programs that involved community group interventions (García-Moreno et al., 2015b), and healthcare systems responses (García-Moreno et al., 2015a) were likely to achieve success. Finally, others recommended engaging men and boys as allies in violence prevention interventions based on gender-transformative approaches (Jewkes et al., 2015).

The Society for Nutrition Education and Health Action (SNEHA) is a Mumbai-based non-governmental organization that has been working toward improving the health and wellbeing of women and children in urban informal communities for 20 years. The SNEHA program on Prevention Violence against Women and Children (PVWC) includes 10 counseling centers across Mumbai, linked with community mobilization, health services, police, and legal support (Daruwalla et al., 2009, 2015). The program has established a network of community-based women volunteers monitoring the safety of women and children and has introduced technology to document cases of violence. Outreach includes group education and enablement with women, men, and adolescents and individual voluntarism (Chakraborty et al., 2017 , 2020). Awareness and understanding of violence and knowledge of rights and resources are developed through group work and campaigns that enable community members to plan individual and collective strategies for primary and secondary prevention.

In 2017, the program initiated a large cluster randomized controlled trial of community mobilization to prevent violence against women and girls in urban informal settlements in Mumbai (Daruwalla et al., 2019b). The primary outcome is reduction in physical and sexual domestic violence after three years, comparing 24 clusters of around 500 households that receive the community mobilization intervention with 24 clusters that do not. The intervention was rolled out in four phases of six clusters each. Intervention areas receive community-based services that include women’s, men’s and adolescent voluntary groups, a cadre of voluntary frontline workers, community campaigns and events, and crisis counseling services which also include legal and medical aid. Control areas receive only crisis intervention and counseling and extended services. A theory of change for the intervention has been developed (Daruwalla et al., 2019a).

Trial clusters are located in large informal settlements (slums) in Mumbai’s central and eastern suburbs. According to the 2011 Census, over 40% of Mumbai’s inhabitants live in slums (Chandramouli, 2011). UN-Habitat (2016, p. 2) defines slums as sites where inhabitants suffer from one or more of the following housing deprivations: access to improved water source, access to improved sanitation facilities, sufficient living area, housing durability, hazardous location, and security of tenure. However, slums are also important informal economy hubs, include diverse and socially heterogeneous settlements, and have been notable sites of civic activism and urban citizenship (Appadurai, 2001).

We had selected clusters in which NGOs or community-based organization were not active or working, and whose communities were identified as vulnerable based on a vulnerability scorecard to assess maternal and child risk. These factors included non-durable housing, illegal or unmetered electricity connections, no private or communal water supply, no communal or private toilets, hazardous locations (dumping ground, polluted water, railway line or airport), and rental accommodation (Osrin et al., 2011).

Accordingly, high vulnerability clusters were located in precarious areas like marshy land, hilly areas, or adjoining polluted rivers or canals, where less than half of houses are concretized (*pukka*) and most residents use public toilets. Residents are usually engaged in the informal workforce, have less security of tenancy and face environmental risks like flooding. In contrast, lower vulnerability clusters are settlements where residents have lived for longer durations (more than 30 years), have well-built material infrastructure and services, security of tenure, and actively participate in civic governance.

After candidate clusters were identified, we entered each community to approach key stakeholders and influential actors for a cluster guardian consent meeting. These included Integrated Child Development Service (ICDS) or *aanganwadi* workers, elected officials or their representatives, members of local voluntary groups (*mandals*), community elders, and members of women’s self-help groups (*bachat gat*). Consent meetings usually involved 25–30 participants. We ensured that these groups were representative of the community in terms of gender (half of them were women) and age (included the young and elderly). We obtained consent from all but two clusters.^1^

## Adapting and Implementing PLA in Prevention of Violence Against Women and Girls

Our decision to use PLA techniques in formative community mobilization draws on our previous experience of using participatory approaches in urban informal settlements after the disastrous July 2005 Mumbai floods, which led to the loss of lives and property in the city and disproportionately affected its most vulnerable populations (Daruwalla & Prevention of Violence against Women and Children, 2012). This “Micro Planning” process was initiated in 2006 by the United Nations Children’s Fund (UNICEF) across vulnerable informal settlements. The objective of this rapid community needs assessment exercise was to enable communities to prepare in advance for disaster management and undertake their own development process through partnerships with government functionaries, local self-governing bodies, and other community stakeholders. The process used PLA techniques like Transect Walks, Timeline and Trends Analysis, Community Resource Mapping, Venn Diagrams, Mobility Mapping, and Matrix Rankings. In urban informal settlements in our primary program area the process reached diverse communities and individuals and strengthened our intervention work. In Dharavi—one of the largest informal communities in Asia and our primary program area—the process reached diverse communities and individuals and strengthened SNEHA’s intervention work.

In the trial, we adapted six PLA techniques from this process to the context of gender relations, gender inequality, and violence against women and girls. The activities were designed to gain an understanding of the situation—particularly experiences of women and girls—with regard to perceived prevalence of domestic violence and violence in public spaces, the situation of gender inequality in the community, preferences for and access to services and support, and their aspirations.

One of our key objectives in adapting PLA techniques to the context of VAWG was to enable women’s and girls’ presence and participation in public spaces, and address their issues and concerns (which were not limited to VAWG). Our collective insight from working on violence prevention over the years informed this approach. We have learnt that interventions are often successful because they destabilize public–private boundaries that consign intimate violence to domestic settings, and open up spaces for women’s participation in public life. At the same time, we adopted the strategy of foregrounding health and wellbeing issues in the PLA exercises to diffuse possible tensions or backlash. Communities were also able to perceive us as service providers who could provide help and support in situations where they had no access to such networks.

Table 1 provides summaries of the six PLA techniques: Timeline Analysis, Conflict Analysis, Safety Mapping, Mobility Mapping, Matrix Ranking, and An Ideal Community (adapted from International Rescue Committee, 2014). We piloted the PLA techniques between March and May 2017, and further refined them along with documentation and reporting guides. We implemented them in the four phases of the trial as follows: Phase One in March 2018; Phase Two in September 2018; Phase Three in January 2019; and Phase Four in February 2019 (see Supplementary Web Table for a detailed timeline).

**Table 1.** Summary and Objectives of PLA Techniques.

PLA Technique	Objective and Implementation
Timeline Analysis	To understand the community’s history and perceived prevalence of violence against women and girls (VAWG) by non-intimate partners in public spaces and domestic violence (DV) in the past, at present, and in the near future. First, participants were asked about how the community was built, how residents acquired services and infrastructure, what were the natural and social calamities they faced and how these were overcome. Next, they were asked about the perceived prevalence of public VAWG and DV ten years ago, at present, and whether it would increase or decrease in the next ten years. These were then represented in the form of a graph.
Conflict Analysis	To understand the community’s perceptions about the predominant or most prevalent causes of familial conflict and how it leads to DV and VAWG. Participants were presented with seven possible causes of family conflict: familial and social norms (concerning women’s and girl’s dressing and mobility); expectations, roles and responsibilities; education and employment; property disputes; financial constraints; medical problems (including mental illness); and addiction. They were asked to assign the highest proportionate percentage to the causes they thought were predominantly responsible for conflict. These were then represented in the form of a pie chart.
Safety Mapping	To understand the community’s perception and experiences of safety from VAWG in public spaces and common resources accessed by women and girls in everyday life. First, participants were asked to draw a map of their neighborhood on a chart paper and then list out locations of common resources and spaces accessed by women and girls (e.g., schools, markets, playgrounds, public toilets). Then, they were asked to rank each of these locations on the basis of how safe women and girls felt during the day and at night. These rankings were represented in the form of stars near the locations on the map (four stars for most safe and one for least safe).
Mobility Mapping	To understand the location and distances of available resources in the community that can be accessed by women and girls in cases of emergencies when they face violence. Participants were first asked to list which resources they thought could provide such assistance. Then, they were asked to plot these on chart paper based on the general geographical direction and distance. They listed distance in terms of time (hours, minutes) and space (meters, kilometers).
Matrix Ranking	To identify community members’ preferences for particular services or resources under specific conditions in contexts of VAWG or DV and evaluate the reasoning and motivations behind these choices. Participants were first presented with the matrix on a paper. This included eight “services” listed across the top row: family, relatives and neighbors; panchayat and community leaders; self-help or women’s groups; ICDS or community health volunteer; police; NGO or community-based organization; private doctor; public hospital. It also included five “conditions” listed in the first column: proximity of service; availability during time of crisis; previous experience with service; fear of private matter becoming public; fear of breaking the family. Participants were presented the vertical “conditions” first and then asked to rank their preference for “services” by assigning a score between 1 (most preferred) and 8 (least preferred).
An Ideal Community	To visualize what life would look like for women and girls in a community where violence, disrespect, and discrimination against them no longer existed; to assess what factors would need to change in order for this vision to become a reality. Participants were informed about the meditative nature of this exercise and then asked to close their eyes and sit in silence while facilitators read aloud a narrative of an “ideal community” where there was no VAWG, no discrimination and women and girls had equality. They were asked to visualize themselves in such a world and then envisage what their existing family and community relationships with would look like: how they could move around, what they could wear, how their relationships with men would be, and what the future would look like for young women and girls in such an ideal world. This was followed by an open discussion.

Each community mobilization cycle consisted of 11 PLA exercises per cluster. With the exception of Timeline Analysis, which was conducted once at the beginning, each activity was conducted twice across multiple locations in the cluster to ensure coverage and participation. As the first activity, we ensured that Timeline Analysis was conducted in a prominent and central location in the cluster. We also ensured that participants in this PLA exercise included key stakeholders who were present during the cluster consent meeting, community members who volunteered to support our team, and community elders. Approximately 25–30 residents participated in each PLA event. While each technique had a central theme that guided discussions, all exercises were interactive and participatory. 6,070 women, men, and adolescents participated in a total of 264 PLA exercises and we identified 66 cases of domestic violence (Table 2).

We conducted ethnographic participant observation to produce contextual data to make process evaluation more robust. We recorded our observations as detailed fieldnotes (Emerson et al., 2007) and conducted short informal interviews with key participants based on a topic guide. Overall, we observed and documented 120 PLA exercises across 24 intervention clusters (Table 2). These observations were transcribed and collated in Microsoft Excel.

**Table 2.** Phase-Wise Distribution of Outreach, Case Identification and Observations Across PLA Exercises.

	Total Outreach	Cases Identified	PLA Exercises Observed
Phase 1	1,152	13	38
Phase 2	1,280	17	31
Phase 3	2,018	23	28
Phase 4	1,620	13	23
Total	6,070	66	120

Summaries of each cluster context were written on the basis of these observations and supplemented by community team reports and socio-demographic data made available from the trial baseline survey. Each entry from the PLA was subsequently coded to generate themes (Saldaña, 2013). These were developed into a context document for the entire intervention area. We have made the PLA data available on the Open Science Framework (OSF) database (Osrin et al., 2020; see Data Accessability Statement). In the next section, we discuss the basic methodological anatomy of PLA exercises, which consisted of three stages: mobilization, facilitation, and dissemination.

### Mobilization

Prior to starting the PLA exercises, the community team (consisting of one officer and three organizers per cluster) mapped cluster boundaries and resources such as ICDS (*aanganwadi*) centers, municipal health posts, ward councilor offices, police beats, public distribution system shops, local community organization or societies, connected with participants who had taken part in the cluster consent meetings, and identified accessible open spaces in the neighborhood where participants could easily gather. These included small fields or open plots of land (*maidan*), large alleyways (*galli*) in front of homes, temple courtyards or *aanganwadi* centers. The team consulted community members to account for women’s daily responsibilities and other factors (e.g. water supply, school hours) and scheduled the time at which to conduct the PLA exercises (usually two PLA exercises were conducted in a day).

The team obtained consent from residents for conducting the exercise and invited them to participate as volunteers. Volunteering was based on the time residents could commit, and usually involved tasks like inviting their neighbors and acquaintances for the PLA exercises and informing them about the program’s services. Generally residents who had participated in the cluster guardian consent meeting and showed active interest in the PLA exercises volunteered their time, even though we informed all attendees about the possibility of volunteering.

After this, the team conducted a 2-day mobilization and rapport-building exercise with community members across the entire cluster. They introduced themselves and program services, provided information about PLA and intervention services, like forming women’s, men’s and adolescent groups, crisis counseling services and support services like police and health systems. On the day of the PLA exercise, the team would focus their mobilization efforts in the *galli* or *mohalla* adjoining the location for the exercise.

### Facilitation

Each PLA exercise was facilitated by one member of the community team, which was decided in advance. One member was assigned documentation and reporting responsibility, whereas the other two mobilized participants and registered them. Once participants had gathered at the location, the facilitator welcomed them, introduced the team, and shared brief information about program services. The facilitator began the PLA exercise by introducing and contextualizing the theme with reference to everyday life in urban informal settlements (e.g., mobility, service preference, safety). While each technique had a central theme that guided discussions, all exercises were interactive and participatory.

After this introduction, facilitators posed questions to participants and encouraged them to actively participate and respond. Facilitators paid attention to ensure that no participant would dominate the discussion (usually men and elderly participants) and invited divergent responses. They also asked follow-up questions to further discussion and elicit in-depth responses and encouraged participants to share their experiences. Facilitators again emphasized the importance of confidentiality in sharing these details and experiences, and discouraged participants from sharing identifiable details (participants were encouraged to approach the team in private after the exercise if they wished to discuss potential incidents of violence or abuse). These discussions were recorded by another team member into the documentation guide.

Once the exercise was concluded, the facilitator introduced the organizer responsible for intervention activities in the cluster. They reiterated the scope of program services like forming women’s, men’s and adolescent groups, crisis counseling services and support and working with the police and health systems. The team also identified participants who were proactive in the exercise and would approach them later to form groups in the neighborhood. Upon conclusion, the team also completed their reporting and documentation. They summarized the key findings from the PLA, briefly described the mobilization process and participant responses, and wrote down short reflexive conclusions after discussing among themselves and consulting their documentation sheets.

In PLA exercises where researchers conducted participant observation, they approached a few key participants—for instance, those who contributed proactively or expressed knowledge and insight about their community—after the exercise concluded and conducted short qualitative interviews based on documentation and observation guides. These included questions on community history; perceived prevalence and causes of domestic violence and violence against women and girls; crime and social disorder; social cohesion and unity; collective mobilization; support for prevention activities; prior experience with NGOs; women’s availability of and access to open spaces. Researchers explained the objective of the interviews and obtained informed consent verbally. These short interviews lasted for 10–15 minutes and involved speaking with two to three participants. Participant responses were recorded into the sheets verbatim along with other observations.

### Dissemination

Dissemination of PLA findings was the final step in formative community mobilization and initiated program services such as group work and crisis counseling activities in intervention clusters. The community team prepared a dissemination plan based on the key findings from their reports, after which they returned to intervention clusters and mobilized key participants and stakeholders for a meeting. Similar to the cluster guardian consent meetings, key stakeholders included ICDS workers, community health volunteers, municipal officials, police officers, and community members who participated in the PLA exercises. The team presented summarized key findings from each PLA technique and invited feedback and discussion. The team then introduced the organizer in-charge of the cluster and the counselor, explained their roles and responsibilities, distributed pamphlets with the program contact details, shared the address of the community center and briefly outlined the process of maintaining confidentiality and consent in crisis interventions.

## Findings

In this section, we present the key thematic findings from each of the six PLA techniques which were synthesized from the community team reports for each cluster as well as from our ethnographic observation and interviews.

### Solidarity, Cohesion and Perceived Prevalence of Violence (Timeline Analysis)

**Figure 1.**
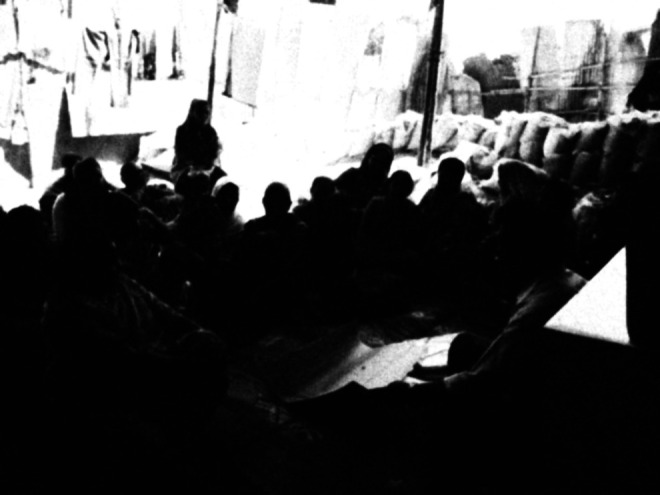
Intervention team conducting a Timeline Analysis PLA exercise in an open space. Photo by: Community intervention team. (Note: Photos have been edited to preserve participant confidentiality).

In the first part of this PLA exercise, community members across almost all clusters spoke about the importance of “unity” (*ekta*) in building and sustaining life in informal settlements. They shared stories of confronting inclement weather and hostile environment while building their homes, as well as enduring adverse social and ecological events, like the 1992–1993 riots and the 2005 floods. Residents shared stories of how they worked with each other and the civic system in such times to obtain essential services like potable water, sanitation and electricity (Figure 1).

Although women’s participation was reported less, we observed some crucial instances of such actions in a few PLA exercises. For instance, in one exercise in a high-vulnerability cluster, women spoke about how they had collectively resisted slum demolitions; whereas in another cluster, women and men said that they had participated in large rallies for slum dwellers’ housing rights. We also observed challenges to fostering ties of unity in a few high-vulnerability clusters. In one cluster, some interview respondents noted that demographic change caused by out-migration of older residents and in-migration of informal workers weakened social ties. In two other heterogeneous low-vulnerability clusters, when we interviewed a few elderly women they mentioned that past social fissures between different communities were caused by competition over limited resources like land, which adversely affected social ties. In one of these clusters, members of one community said they did not go to the other side as a result of this.

**Figure 2.**
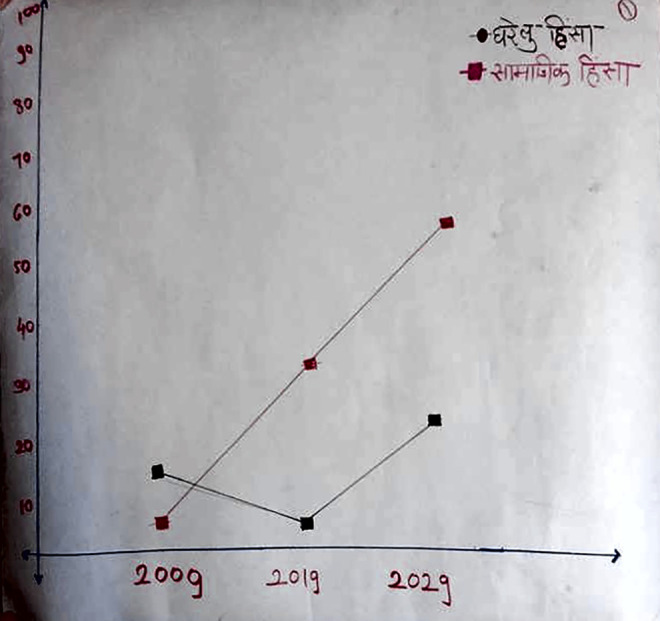
Photograph of a Timeline Analysis PLA exercise chart depicting perceived prevalence of public VAWG (first on the top-right corner) and domestic violence (second on the top-right corner). Photo by: Community intervention team. (Note: Photos have been edited to preserve participant confidentiality).

In the second part of this PLA exercise, we observed that in almost half the clusters, women normalized, if not entirely denied, the prevalence of domestic violence. In one PLA exercise, a woman even suggested that “This is the story of every house” (*har ghar ki kahani*) (Figure 2). In the discussions which took place in these exercises, women generally contextualized domestic violence as a part of marital relationships and took place when “normal” conflicts escalated. Although collective action on violence was generally less frequent across all clusters, in our discussions community members openly spoke out and took action against public violence. These were linked to forms of everyday violence and vulnerability such as substance abuse, addiction, crime, and lack of infrastructure like streetlights and public toilets. In low vulnerability clusters, community members generally said that they dealt with perpetrators of public violence using physical force. In contrast, when we interviewed residents—both women and men—in high vulnerability clusters, they reported that “wrongdoers have [more] unity,” and rely on their friends and relatives to support them. We term this negative solidarity, which included threats of harm or intimidation that prevented people from speaking up or organizing collectively, as well as a tendency among detractors to close ranks, discourage others, or criticize and mock them.

### Gender, Inequality and Unpaid Socially Reproductive Care Work (Conflict Analysis)

**Figure 3.**
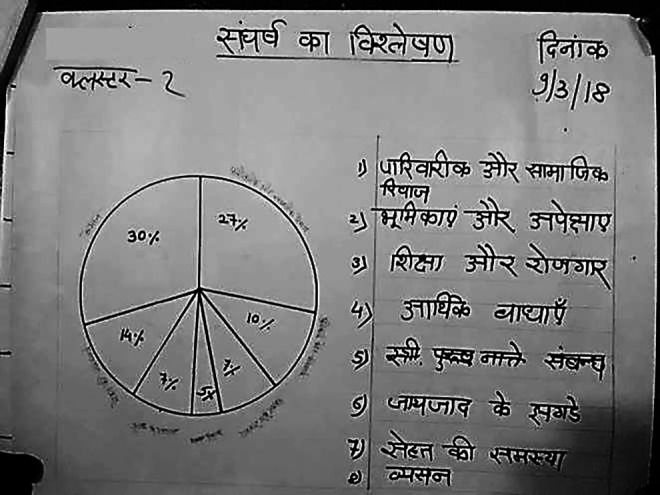
Photograph of a Conflict Analysis PLA exercise chart depicting the main sources of household conflict (the list on the right: familial and social norms; roles and responsibilities; education and employment; financial constraints; property disputes; health issues; addiction). Photo by: Community intervention team. (Note: Photos have been edited to preserve participant confidentiality).

Women said that the predominant sources of conflict (*sangharsh*) lay in familial and social norms, women’s roles, responsibilities and expectations, and women’s and girls’ education and employment (Figure 3). Their discussions—which we observed in the exercises themselves—on the nature and causes of conflict underscored the centrality of unpaid socially reproductive care work and how it devalued them, normalized conflict and suffering, and led to violence and abuse. We observed that, although women bore a disproportionate brunt of conflicts and were often blamed for them, some participants justified such pressures and even suggested that conflicts could be prevented if women conformed to norms. Accordingly, the onus was on women to be selfless (*niswarth*) and use understanding (*samajh*) to resolve conflicts.

However, in the ensuing discussions, many participants upended such gender unequal connotations and emphasized that it was their labor that sustained families. In one of the PLA exercises, a group of women differentiated between internal and external causes of conflict: internal conflict could still “be managed,” but external conflict, like poverty or health issues, could not. A group of Muslim women in another cluster brought up the issue of how pressures of gender unequal norms and religion intersect and cause suffering within and outside the community.

### Space, Vulnerability and Everyday Violence (Safety Mapping)

**Figure 4.**
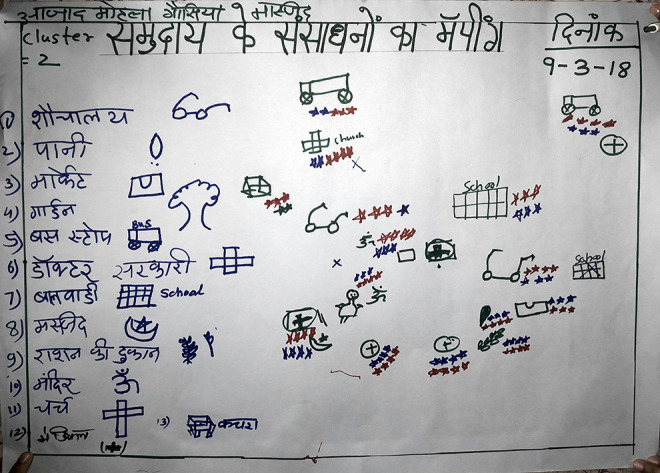
Photograph of a Safety Mapping PLA exercise chart depicting public spaces in the community and how safe they are (the list on the left: public toilet; public tap; market; garden; bus stop; govt. healthpost; ICDS center; mosque; public distribution shop; temple; church; pharmacy).Photo by: Community intervention team. (Note: Photos have been edited to preserve participant confidentiality).

Drug addiction and substance abuse were identified as major issues threatening the safety of women and girls, as users often resorted to violence and harassment in public. In the discussions that ensued in the exercises, participants explained that these were further exacerbated by absence of civic infrastructure like functioning toilets and street lights, especially in high vulnerability clusters (Figure 4). When we interviewed women and girls, they said that as a result of such vulnerabilities their mobility was restricted by families to protect their “honor” (*izzat*) and they were often blamed for inviting trouble. However, in many discussions we observed that young women and girls often countered victim-blaming narratives by sharing and critiquing their experiences of violence, which included incidents of verbal sexual harassment, molestation and stalking. For instance, in one exercise, when an elderly participant said that men only “bother a girl who is wrong (*galat*)” a group of young women and girls countered her, and said, “It is not our role to pass judgment on a woman’s character.” In contrast, factors such as the presence of known people and acquaintances in public spaces, well-lit lanes and strong social and physical boundaries and familiarity among residents contributed toward safety. Particular neighborhoods (*mohalla*) or alleyways (*galli*) which were considered safe were thought to be extensions of the private or domestic space.

### Constrained Mobility and Access to Services (Mobility Mapping)

**Figure 5.**
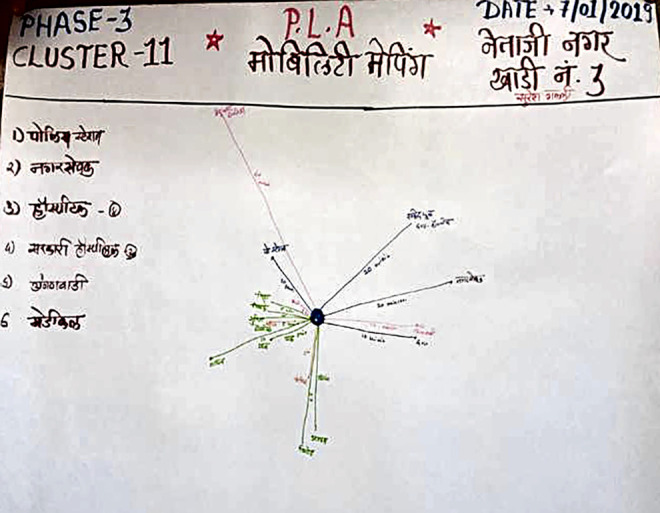
Photograph of a Mobility Mapping PLA exercise chart depicting resources that survivors of VAWG can access and their accessibility (the list on the left: police station; ward councilor’s office; private hospital; public hospital; ICDS center; pharmacy). Photo by: Community intervention team. (Note: Photos have been edited to preserve participant confidentiality).

Women’s mobility and access to health and support services were shaped by the material conditions of the clusters, the availability of services and transport facilities, and their awareness, knowledge, and skills (Figure 5). For instance, in clusters that were sequestered and located peripherally, we observed that women’s mobility was constrained by significant transport expenses, as well as the risk of facing sexual harassment and violence—exacerbated by seclusion and lack of infrastructure. Women who lived in centrally-located clusters with nearby bus stops and main roads reported that these factors enabled their mobility.

Apart from physical or material constraints, lack of awareness about services also constrained mobility. In the exercises, many women reasoned that they would not be able to access services like municipal ward councilor offices, health posts, and police posts even if these were located in the vicinity, as they had simply never accessed them before. In addition to this, families also controlled women’s mobility, often citing the risk of public sexual violence. In one exercise, a group of women voiced the concern that if survivors approached such services it would threaten confidentiality as “word would spread” (*baat fail jayegi*), whereas others said that prevalent social norms prevented women from going out in public, which was seen as the responsibility of men. We observed that women who worked reported a higher degree of mobility as well as an awareness of services and helplines, suggesting that education and workforce participation have positive effects on mobility.

### Trust, Support and Services (Matrix Ranking)

**Figure 6.**
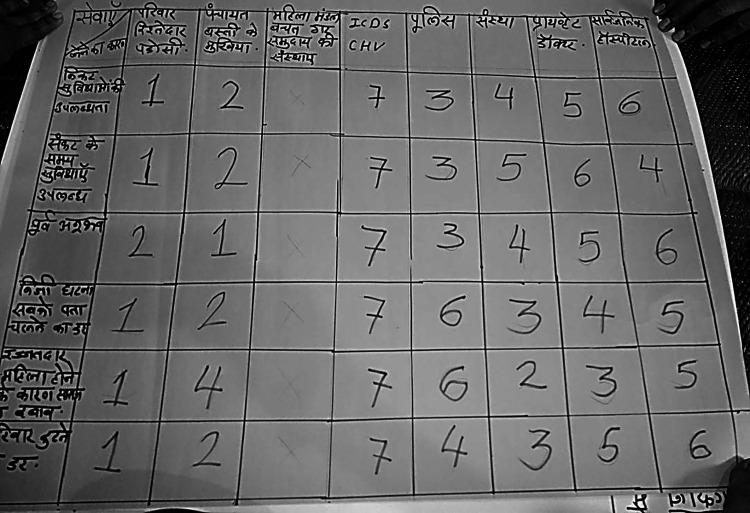
Photograph of a Matrix Ranking PLA exercise chart depicting service preference (See Table 1 for the detailed list of services and conditions). Photo by: Community intervention team. (Note: Photos have been edited to preserve participant confidentiality).

Women expressed most preference for family, neighbors and relatives, with many even suggesting that they would call on their neighbors before relatives (Figure 6). Involving the police in such matters would bring dishonor (*beizzati*). However, others expressed caution about family and community-based services, like the *jamaat* or *panchayat*, as they could pressure survivors to compromise, in which case they noted a preference for services which were neutral. Such organizations were exclusively male and largely rejected legal recourse for survivors of violence in favor of maintaining the familial status quo. And as these had more cultural legitimacy, many young women also faced internalized pressure to accept their decisions. Thus, the onus to find a solution for the domestic violence situation was on women themselves. In comparison, police or NGOs were “neutral” as they were located outside communitarian structures and operated on legal principles.

However, we found that women’s existing relationships with police, public health institutions, and other community-based organizations were limited and, in many clusters, non-existent. In high vulnerability clusters, women often mentioned in our interviews that police were involved with those engaging in criminal acts and violence. We observed stronger linkages between community women and services in low vulnerability clusters, where they had well-established relationships with municipal ward councilor’s offices and political party functionaries.

Women participants in only seven clusters across Phases 3 and 4 reported that they were aware of a women’s organization in the vicinity, which was based out of a local school. Despite this, community women were generally unaware of how NGO services functioned, and expressed skepticism. After our discussions during the exercises, they felt that women service providers (like NGO workers) could be beneficial because “women would only understand women’s needs” (*aurat hi aurat ki zaroorat ko samajh sakti hain*). In one such exercise in a high-vulnerability cluster, some women reasoned that our presence in the area and the participatory nature of our interaction demonstrated our resolve. An elderly woman even remarked, “If you have come here, you will do something for us!”

### Hope, Tensions and the Future (Ideal Community)

**Figure 7.**
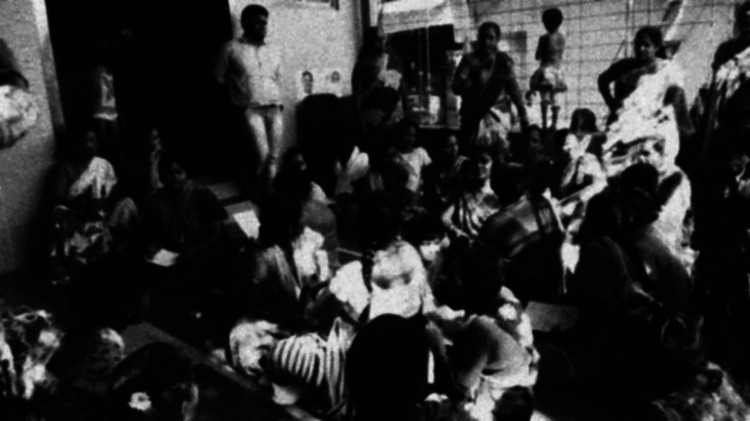
Intervention team conducting An Ideal Community PLA exercise in a Buddha Vihara. Photo by: Community intervention team. (Note: Photos have been edited to preserve participant confidentiality).

Women living in high vulnerability clusters generally found it difficult to imagine an “ideal community,” as they were unable to look beyond the present issues and problems that confronted them (Figure 7). Although their visions of an ideal community were limited to their immediate neighborhoods, they did express hope and desire for change, especially for future generations of women and girls. In one PLA exercise in a high-vulnerability cluster, young women and girls expressed expectations that their parents—especially mothers—would stop discriminating between them and boys and divide responsibilities and privileges equally. In the same exercise, another young woman shared her vision of supporting survivors to help them realize their “freedom” (*aazadi*). Other aspirations, shared in the PLA exercises by young, married women, were more personal and intimate. One participant envisioned a reduction in burden of care; whereas, in a different exercise, another woman wished for mutual understanding in intimate relationships based on “love and equality.” A few middle-aged women mentioned in a different exercise that men should accept their mistakes and “run the household together,” instead of consuming tobacco, cigarettes, and alcohol—which was “cutting their life in half.”

Across multiple exercises, we observed that participants generally envisaged two divergent mechanisms of change: the first emphasized women’s and girls’ responsibility to shouldered the burden of change by possessing “proper knowledge” (*sahi jankaari*) as we “cannot control the environment” (*mahaul*). The second perspective challenged this and stressed that others had to first change their views as this created the environment (*mahaul*) in the first place.

## Discussion

Our experience of developing and implementing PLA techniques in a formative community mobilization process demonstrated its promise as a collaborative and polysemic exercise which draws on core principles and methods of participatory approaches. Our overall experience shows that community mobilization combined with affective engagement as well as rigorous methodologies of documentation and reflection can build relations with communities and yield contextual and nuanced data.

We found existing mechanisms of mobilization and support among communities in urban informal settlements, articulated through notions of “unity.” This corresponded inversely with cluster vulnerability, as lower vulnerability clusters reported stronger social ties. In contrast, we observed an emergent phenomenon we describe as “negative solidarity” in high vulnerability clusters, which is a potential barrier to unity and collective action. Despite this, using participatory approaches opened possibilities of engaging with community members who perceived NGOs as potential support structures and articulated concerns and expectations.

We also demonstrated the flexibility of participatory approaches by adapting PLA techniques to the context of violence against women and girls and gender inequality. Even though many participants denied or normalized domestic violence, multiple focal points of PLA techniques and the possibility of facilitating them in an open-ended manner—which was also responsive to community concerns—led to critical discussions among participants about their understandings, experiences, and perceptions of violence.

For instance, despite initial reluctance to disclose perceived prevalence, women and girls spoke of violence and its relation to other forms of inequalities more openly in PLA exercises like Conflict Analysis and Safety Mapping. Similarly, even as women and girls spoke about factors which contributed to feelings of safety and security, their experiences and responses in PLA exercises like Mobility Mapping and Matrix Ranking showed that there were numerous constraints posed by social norms, attitudes and community relations when it came to accessing services. Our synthesis of evidence also demonstrated that perceived prevalence of gender violence was linked to structural factors such as crime and everyday violence, as well as to the disproportionate burden of unpaid reproductive care work on women.

Further, as our facilitation of PLA exercises was closely attuned to women’s daily routines, it contributed to creating new spaces for discussion and reflection and destabilizing rigid public–private boundaries. Our processes of data collection, analysis, reflection, and interpretation were also done collaboratively between researchers and fieldworkers during the community mobilization and reporting phases. This helped us gain a deeper understanding of factors that constrain or enable community women’s pathways to access care and support. Even though resources like NGOs or CBOs were not present in clusters, participants perceived NGOs as potential support networks and services, though not only with regard to violence. They underscored how gender and feminization of labor and everyday life mediated their preferences by emphasizing values like empathy, understanding, confidentiality, and neutrality.

Our recent observations showed that PLA exercises had played an important part in subsequent community mobilization processes for forming voluntary groups with women, men, and adolescents in intervention clusters. Many women who had participated in the exercises showed an interest in joining groups. We found that PLA exercises helped community women engage in conversations about their private lives and experiences of abuse and violence with our community team members, most of whom were also women, and were aware about these issues and could potentially help them as well. This paved a way to shaping relationships between women and the program.

Moreover, as our mobilization efforts also involved community health volunteers and *aanganwadi* workers, we were able to strengthen the linkages between these services and the communities later on. For instance, we identified 66 survivors of violence while implementing PLA exercises across all four phases (Table 2). In the following period between the conclusion of PLA exercises and dissemination meetings, 31 women from intervention clusters had accessed crisis counseling services. In clusters where subsequent community mobilization efforts faced challenges or difficulties, community team members were supported by women who had participated in or volunteered during the PLA exercises. This helped in maintaining the momentum of the intervention.

These findings and learnings are particularly relevant when we consider help-seeking patterns among women who face violence. According to NFHS-4, only 29% of women who have faced *physical and sexual violence* and 14% of women who have faced *physical or sexual violence* had sought help, with the most common sources of help being the natal family (65%) and marital family (29%). Police services (3%) and social service organization, lawyers, and medical personnel (1%) were least sought sources of help (IIPS & ICF, 2017, p. 572).

In such contexts, participatory community mobilization exercises which address violence against women and girls are effective and sustainable strategies to gain access to communities and involve their most vulnerable and marginalized members in the process of collective mobilization and change, thereby unsettling dominant social hierarchies (cf. Mosse, 1994). For instance, ethnographies of gender violence in urban informal settlements have shown that everyday pressures of vulnerability and poverty constrain women’s ability to seek support from “outsiders” like NGOs (Datta, 2016), as communities differentiate between public and private forms of violence (Ghosh, 2011). In such cases, women often rely on networks of informal support (Snell-Rood, 2015), and even critically consider the relationship between gendered inequalities and violence (Roy, 2003), which further informs their ability to negotiate violence, discrimination and inequality (Chakraborty, 2020).

As our evidence showed, after participating in PLA exercises, women and girls were more receptive to NGOs, as they perceived such services as accommodating women’s needs (e.g., confidentiality), as well as sources of care, solidarity, and support (e.g., women service providers). Here, we see the promise of participatory approaches, as they work toward *aligning* program processes and outcomes with the needs and expectations of community women through dialogic, open-ended exercises. This opens up new spaces of engagement for women and girls, who continue to face serious and subtle forms of social exclusion. The practical nuances of PLA are salient as well, as discussing mundane or everyday concerns such as household work, safety, access to services and so forth, is a crucial buy-in. This also presents opportunities to connect women and girls to both non-governmental and state services—relationships that were non-existent in many high vulnerability clusters.

We are currently using our PLA findings along with survey data to develop program evaluation using a critical realist case study design (Pawson & Tilly, 1997; Yin, 2009). Embedding ethnographic participant observation in program design enabled us to gain grounded understandings of social context, select clusters as candidate cases, and uncover potential causal mechanisms which can lead to our hypothesized program outcomes.^2^

### Limitations

We used PLA techniques for formative community mobilization and rapid needs assessment with particular emphasis on the question of violence against women and girls and gender inequality. This paper is unable, therefore, to address the scope of adapting or scaling it to different social contexts or issues, as this would require program-specific reflections on prioritizing themes and questions, rather than a set of standard guidelines (apart from the core principles of participatory approaches). Although we tried to ensure that our outreach was inclusive and representative of the community in terms of religion, caste, age and disability, it is likely that women and girls from more marginalized and vulnerable positions were unable to participate in PLA exercises or disclose their experiences and insights in public meetings.

## Conclusion

Over the last decade, interventions to prevent violence against women and girls have increasingly used community mobilization strategies for outreach, advocacy, and intervention (García-Moreno et al., 2015b). Our experience of adapting PLA shows the promise of using participatory approaches in such interventions. Our findings suggest that participatory approaches help build relationships and networks with community members and support services in vulnerable urban informal settlements, and generate nuanced understandings of violence, gender inequality and potential mechanisms and barriers which can aid interventions. We found that community members hold multiple, and at times contradictory, perspectives on interventions to prevent violence. The promise of participatory approach lies in giving voice to the marginalized and vulnerable, like women and girls, and creating new spaces of interaction and engagement. In such contexts, the use of participatory approaches—which also help programs understand the community’s preparedness—is particularly important because a community that is aware of and responsive to its problems is more likely to invest in its own development and wellbeing. This forms a crucial infrastructure upon which programs can adapt, grow and engender change and transformation.

## Supplemental Material

Supplemental Material, supplementary-web-table - Using Participatory Learning and Action in a Community-Based Intervention to Prevent Violence Against Women and Girls in Mumbai’s Informal Settlements Click here for additional data file. Supplemental Material, supplementary-web-table for Using Participatory Learning and Action in a Community-Based Intervention to Prevent Violence Against Women and Girls in Mumbai’s Informal Settlements by Proshant Chakraborty, Nayreen Daruwalla, Apoorwa Deepak Gupta, Unnati Machchhar, Bhaskar Kakad, Shilpa Adelkar and David Osrin in International Journal of Qualitative Methods

## Notes

1 In the first cluster, community members suggested that there was no need for interventions on VAWG as they claimed there was no violence in their neighborhood. In the second cluster, residents refused consent after raising objections about the sensitive nature of the topic and were opposed to women openly discussing their private affairs with outsiders.

2 We understand causal pathways as hypothesized context-mechanism-outcome configurations (CMOCs), as suggested in realistic evaluation (Pawson & Tilly, 1997). We have identified causal pathways at the individual, group and community levels—the socio-ecological (Heise, 1998) units at which program interventions are delivered. We propose that in different cluster contexts (C), the intervention activates underlying mechanisms (M) of change—for instance, expansion of reference groups, challenging unequal norms and behaviors and collectivism—and thus leads to program outcomes (O) of primary and secondary violence prevention.
