# Supplementary material for: Using Participatory Learning and Action in a Community-Based Intervention to Prevent Violence Against Women and Girls in Mumbai’s Informal Settlements
Source: Int J Qual Methods. 2020 Nov 24;19:1609406920972234. doi: 10.1177/1609406920972234 (PMC8995557; doi:10.1177/1609406920972234)
Supplement: Supplemental Material, supplementary-web-table - Using Participatory Learning and Action in a Community-Based Intervention to Prevent Violence Against Women and Girls in Mumbai’s Informal Settlements [file supplementary-web-table.pdf]

Timeline of developing and implementing PLA techniques in the SNEHA-TARA trial

Timeline	Activity
Mar 2017	The PVWC programme held a one-day workshop on adapting PLA techniques to the issue of violence against women and girls at its centre in early March. Facilitators (ND, BK) shortlisted six PLA techniques for adaptation. Later, the community team conducted mock sessions with the adapted exercises, following which the final structure and objectives were outlined. PC, ND, and BK then refined these into preliminary PLA exercises. PC, ND and BK trained the community team, who then implemented PLA as part of formative community mobilization and rapid needs assessment in one pilot cluster. PC also developed the PLA reporting and documentation guides.
April 2017	PC, BK and the community team collated the community mobilization results and findings and prepared a report for the pilot cluster. Following this, the community team conducted a dissemination campaign in the cluster. PC revised some of the PLA techniques for clarity and cohesion.
May 2017	The community team along, with BK, conducted community mobilization in the second pilot cluster using the revised PLA techniques.
Jun 2017	PC, ND, BK and DO collated the results and findings from both clusters into a final report. The community team

	conducted a dissemination campaign in the second cluster. PC and AG then revised the PLA techniques and collated them into a draft PLA manual with documentation and reporting guidelines.
Mar-Apr 2018	We included the sixth and final PLA technique, “An Ideal Community” (International Rescue Committee, 2014), in the manual and finalized the documentation guide for qualitative researchers. PC, ND, AG, UM and BK trained the Phase 1 intervention team over a two-day training workshop in early March. The intervention team implemented the PLA exercises between mid-March and mid-April across six intervention clusters. They were supported by UM and BK. PC and AG conducted qualitative data collection and participant observation.
May-June 2018	The community team finished reporting and conducted the dissemination campaign in Phase 1 clusters by the third week of May. PC and AG compiled their qualitative data and ethnographic observations into the database and wrote cluster summaries by mid-June. They made minor revisions to the PLA manual to ensure smooth delivery.
Sep-Oct 2018	We trained the Phase 2 intervention team in early September and implemented PLA exercises in six intervention clusters between mid-September and end of October. PC conducted ethnographic fieldwork and qualitative data collection in the clusters, while UM and BK

	observed the meetings and provided support to the team.
Nov-Dec 2018	The Phase 2 intervention team completed their documentation and reporting and disseminated PLA findings in the clusters by November. PC compiled the ethnographic and qualitative data into the MS Excel database. We recruited and trained the Phase 3 and 4 intervention team in December.
Jan 2019	We implemented PLA exercises in six clusters in Phase 3 in January. PC conducted ethnographic participant observation and qualitative data collection in all six clusters, while UM and BK observed meetings and provided support to the team.
Feb-Mar 2019	We implemented PLA exercises in six clusters in Phase 4 from the beginning of February to the first week of March. PC conducted ethnographic participant observation and qualitative data collection in all six clusters, while UM and BK observed meetings and provided support to the team.
April-May 2019	Phase 3 and 4 intervention teams disseminated PLA findings in their respective clusters. PC compiled and transcribed the ethnographic and qualitative data into the MS Excel database and completed the cluster summaries.
Jun-Aug 2019	After discussing the cluster summaries with the research team, PC coded the qualitative and ethnographic data with assistance from AG. The analysis generated themes which corresponded to the PLA techniques and observation guide,

but also included unanticipated and emergent themes. PC wrote the findings into a thematic trial cluster context document covering all 24 intervention clusters. ND, AG, UM, BK, SA and DO reviewed the document and provided feedback. In addition, PC and AG examined prevalence, vulnerability and social cohesion indicators from the baseline survey data and conducted FGDs with the intervention team and preliminary fieldwork to augment the PLA findings. This gave insights regarding how certain clusters were responding to the intervention (e.g., group formation, action, identifying survivors and referrals). All authors discussed these findings and selected eight clusters as candidate cases for the trial evaluation case study design.
